# Pedagogy in practice: a qualitative analysis of evidence-based teaching methods used by graduate-entry near-peer medical educators

**DOI:** 10.3389/fmed.2026.1757648

**Published:** 2026-02-27

**Authors:** Ross Davey, Ana L. S. Da Silva, Marcela Bezdickova

**Affiliations:** 1 Faculty of Medicine, Health and Life Sciences, Swansea University, Swansea, United Kingdom; 2 Cwm Taf Bro Morgannwg University Health Board, NHS Wales, Abercynon, United Kingdom

**Keywords:** clinical educators, education training and development, graduate entry medicine, near-peer education, pedagogy, qualitative, teacher techniques, thematic analysis

## Abstract

**Introduction:**

Near-peer teaching (NPT) has become a fundamental component of modern medical education, theoretically supported by principles of social and cognitive congruence. While the benefits for both learners and educators are well-documented, the actual pedagogical craft including the specific teaching strategies and philosophies employed by Near-Peer Educators (NPEs) remains largely unexamined. This study aims to explore this pedagogical perspective within a cohort of Graduate-Entry Medicine (GEM) students delivering foundational anatomy and physiology teaching.

**Methods:**

This study employed a qualitative research design utilizing reflexive thematic analysis of retrospective written reflective accounts. Participants were GEM students employed as Senior Teaching Assistants at Swansea University Medical School. Over two academic cohorts (2022/23 and 2023/24), NPEs completed a brief ‘Clinical Educators Programme’ and submitted reflective essays on their teaching experiences. A total of 82 essays were analyzed to identify emergent themes regarding the NPEs’ teaching practices.

**Results:**

Near-Peer Educators engaged in a sophisticated pedagogical craft characterized by two central, interconnected themes. They employed a Cognitive Toolkit wherein they deliberately applied evidence-based cognitive science principles such as retrieval practice, dual coding, and concrete examples to manage cognitive load. A second theme of the development of a Humanistic Framework to foster a supportive learning environment was also identified. This included the active establishment of psychological safety, the use of universal design, and adaptive teaching strategies to prioritize learner wellbeing and inclusivity.

**Discussion:**

The findings characterize GEM NPEs as reflective practitioners who move beyond intuitive teaching to blend the science of learning with a deeply relational, humanistic approach. The use of the Cognitive Toolkit demonstrates the efficacy of brief formal pedagogical training, while the Humanistic Framework suggests that NPEs actively construct social congruence rather than relying solely on inherent peer traits. Furthermore, the latter mirrors the values of relationship-centered clinical care. We conclude that NPE training should evolve from instruction in techniques to the development of communities of practice. NPE training should be reconceptualized not merely as academic preparation, but as a foundational element of clinical training that develops medical professionals and educators who are as relational as they are technical.

## 1 Introduction

Near-peer teaching has transitioned from a novel intervention to a fundamental component of the modern medical curriculum. Its widespread adoption is rooted in a compelling theoretical framework that explains its effectiveness for both learner and educator. For the learner, the benefits are largely attributed to the principles of social and cognitive congruence. Social congruence, as articulated in the seminal work by Lockspeiser et al. (1) refers to the reduced hierarchical distance between the student and their near-peer tutor. This flattened power dynamic is thought to create a psychologically safer learning environment, one in which learners feel more comfortable asking “stupid questions” admitting confusion, and engaging in active, exploratory dialog without fear of judgment (2, 3). This increased interaction is a critical facilitator of deeper learning (4).

Complementing this is cognitive congruence, the concept that NPEs, being only slightly more advanced, share a similar knowledge framework and language with their learners (5). Having recently navigated the same conceptual hurdles, they are uniquely positioned to anticipate common difficulties, deconstruct complex topics into relatable parts, and offer analogies and explanations that resonate with a novice’s understanding. They can effectively act as a bridge between the learner’s current knowledge state and the more abstract, consolidated knowledge of an expert faculty member (6).

For the educator, the primary mechanism of benefit is the ‘protégé’ effect, a phenomenon where the act of preparing to teach a subject results in deeper and more durable learning for the teacher themselves (7). The process of deconstructing, organizing, and planning to explain complex material forces the NPE to confront gaps in their own knowledge and to synthesize information at a higher cognitive level (8). Beyond this academic benefit, the role of an NPE serves as a crucial incubator for professional identity formation. It provides a structured opportunity to practice and develop core professional competencies such as communication, leadership, and empathy, skills that are directly transferable to future clinical practice (9). Recent qualitative studies have suggested that this experience is a powerful catalyst for developing a more reflective and conscious professional self (10).

Despite this robust theoretical foundation, a significant gap persists between understanding *how* NPEs implement teaching strategies to formulate effective practice. The actual pedagogical craft of the NPE often remains an unexamined “black box.” This is a critical omission, as teaching is a demanding professional skill. The NPE role is fraught with its own set of well-documented challenges, including role ambiguity, the stress of an increased workload, and significant anxieties about competence that can trigger or worsen the imposter phenomenon (11–14). Compounding this is the widespread inconsistency in NPE training, which is often brief, informal, or absent altogether (15, 16).

This inquiry directs its focus toward a cohort uniquely positioned to possess a more developed pedagogical perspective: students in Graduate-Entry Medicine (GEM) programs. These individuals enter medical school with the academic maturity of a prior degree and a diversity of life experiences. At Swansea University, these GEM students deliver foundational anatomy and physiology teaching across multiple health science disciplines. This study, therefore, aims to look inside the “black box” of this near-peer instruction.

## 2 Materials and methods

### 2.1 Setting

The NPT program at Swansea University’s Faculty of Medicine, Health and Life Science comprised of GEM students holding a formal, paid employed role as a Senior Teaching Assistant. NPEs were recruited through a voluntary application via formal institutional human resources processes. Eligibility required successful completion of prior anatomy examinations; no additional academic ranking criteria were applied. Applicants were asked to outline their interest in anatomy and medical education and were subsequently appointed to posts upon shortlisting process of submitted applications.

The NPEs delivered small-group anatomy and physiology teaching across multiple undergraduate health science programs, typically teaching groups of approximately 6–10 students per group/workstation. Teaching took place in human anatomy laboratory spaces and was embedded within the formal curriculum of the learners’ degree programs, aligned with faculty-defined learning outcomes. The undergraduate modules followed a blended instructional format, consisting of lecturer/senior academic and clinician-led lectures introducing core theoretical concepts, followed by small-group practical workshops utilizing anatomical models and cadaveric prosections to support applied learning. Learning outcomes required learners to demonstrate anatomical knowledge and understanding at a level equivalent to, or below, the curricular expectations of the NPEs’ own medical training.

The NPEs were allocated to teaching posts with a minimum of 2 weeks’ preparation time prior to delivery. Learner attainment of module content was assessed through summative anatomy spotter examinations and/or written examination questions, depending on the program of study.

In preparation for their teaching role, all NPEs completed mandatory training in evidence-based pedagogical principles through a brief, extracurricular ‘Clinical Educators Programme’. NPEs taught with a high degree of autonomy, with session content guided by predefined learning outcomes, within a supported teaching environment. Faculty staff retained responsibility for curriculum design and overall oversight, and academic or postgraduate staff were available during teaching sessions as required, particularly where necessary for safety or specialist support. NPEs were not involved in setting or grading summative assessment of learners.

### 2.2 Study design

This study employed a qualitative research design, utilizing a reflexive thematic analysis of retrospective written reflective accounts. This interpretivist approach was selected for its capacity to facilitate an in-depth, inductive exploration of the participants’ subjective experiences, perceptions, and the meanings they ascribed to their roles as near-peer educators (17). This methodology allowed for a rich understanding of the complex interplay between teaching opportunities and the development of professional identity among medical students. The use of written reflections specifically enabled participants to articulate their perspectives on the benefits of near-peer teaching for both tutors and tutees, as well as the challenges encountered during the teaching process, contributing to a holistic understanding of their experiences (18).

### 2.3 Participants

The participants included in this study were Swansea University Anatomical Senior Teaching Assistant NPEs who have submitted reflective accounts of their time as an educator to the Clinical Educators Programme. The only exclusion criterion were test submissions made by system administrators and blank or non-serious attempts at reflective accounts. A breakdown of available characteristics of participants is presented below in Table 1. The study utilized retrospective analysis of pre-existing written reflective accounts that had been voluntarily submitted by students as part of an educational program prior to the conception of this research.

**Table 1:** Gender and year of study demographics for each cohort of participants.

Cohort	Total (*n*)	Male (*n*)	Female (*n*)	Year 1 (*n*)	Year 2 (*n*)	Year 3 (*n*)	Year 4 (*n*)	Prior teaching experience
2022/23	39	46% (18)	54% (21)	62% (24)	33% (13)	5% (2)	0% (0)	15% (6)
2023/24	43	49% (21)	51% (22)	51% (22)	37% (16)	12% (5)	0% (0)	30% (13)
Both	82	48% (39)	52% (43)	56% (46)	35% (29)	9% (7)	0% (0)	23% (19)

The ethics committee confirmed the sound use of these data without additional consent on the basis that all submissions were anonymized prior to analysis, no identifiable information was retained, and the research posed minimal risk to participants. Data use was therefore consistent with institutional policy on secondary analysis of student-generated material from mandatory part of the module for research purposes.

### 2.4 Clinical Educators Programme

All Near-Peer Educators completed a non-credit-bearing, asynchronous Clinical Educators Programme delivered via the institutional virtual learning environment Canvas (19). The program was designed based on a Teaching, Learning and Assessment module of the MSc in Medical Education to provide explicit training in evidence-based pedagogical principles and is aligned with the UK Professional Standards Framework to support applications for Associate Fellowship of the Higher Education Academy.

The program was delivered longitudinally across the academic year and comprised three phases. Phase 1 (Foundations of Practice), completed prior to any formal teaching, introduced evidence-based principles of learning relevant to clinical anatomy education, including cognitive load theory, retrieval practice, dual coding, and the use of concrete examples. Learning materials included seminal papers, structured checklists, and six short instructional videos (3–22 min), with formative online quizzes to support consolidation (approximately 3.5 h engagement).

Phase 2 focused on peer observation of teaching, supported by short video guidance, selected literature, and a structured formative feedback tool (approximately 2 h). Phase 3 emphasized reflective practice, including a podcast and guided reflection prompts, culminating in submission of a written reflective account between 250–500 words (approximately 3 h). Completion of the reflective essay signified completion of the program. Further illustrative materials are provided in Supplementary Material 1.

### 2.5 Data collection

As part of the Clinical Educators Programme, all NPEs were encouraged to submit a voluntary reflective essay of approximately 250–500 words via the Canvas (19) virtual learning environment. The essays were guided by a series of prompts designed to elicit rich reflection on their experiences, including: “Something difficult and/or unexpected and how you dealt with it” and “Something you have learned from being an anatomy demonstrator”. Submissions were downloaded anonymously from Canvas as PDF files and assigned a numerical identifier to maintain participant confidentiality as per accepted practice (20, 21). Blank or spurious submissions were removed at this stage. Anonymized data was stored on a faculty OneDrive (22) account with restricted access.

### 2.6 Data analysis

Datasets were exported into NVivo 14 (23) to facilitate qualitative analysis. A line-by-line reflexive inductive thematic analysis was conducted following the six-phase framework outlined by Braun and Clarke (24). This iterative and recursive process involved:

a) Data familiarization through repeated reading
b) Open systematic, inductive coding of the entire dataset
c) Axial coding for generation of initial themes from collated codes
d) Selective coding through a two-level review and refinement of themes against the coded extracts and the full dataset
e) Final definition and naming of themes
f) Producing the report (manuscript write-up)

The analysis was conducted by two researchers (RD and MB) who followed accepted practice to reduce the prevalence of bias and engaged in regular dialog to ensure analytical rigor and a rich interpretation of the data (25). Analysis output data was exported to Microsoft Excel (26), for tabularization. Two themes were elucidated from analysis of relevance to pedagogical practice.

No claims of data saturation were made. Saturation is commonly associated with approaches that assume meaning can be exhaustively captured through iterative sampling. In contrast, reflexive thematic analysis adopts an interpretivist stance in which themes are understood as situated, partial, and open to further interpretation. Accordingly, this analysis prioritized interpretive depth and theoretical coherence within a bounded dataset, rather than thematic exhaustiveness.

### 2.7 Research team and reflexivity

The research team comprised of two tenured academics and a postgraduate student. ALSDS (PhD) has an expertise within clinical education and a strong background in qualitative and mixed methods research. She is the program director for medical education courses and has extensive experience medical education research. ALSDS worked with RD to lead the creation of the clinical educator’s program. MB (PhD) is a senior educator in anatomy and oversees the NPT program within Swansea University medical School. RD (MBBCh, MSc) was once a NPE within the program as a medical student, providing him with a strong rooting in the context of the study. He completed a thesis on this topic as part of his postgraduate qualification. RD had limited training in qualitative research methodology prior to this study, but was supported by co-authors. Both MB and RD had personal experience with the NPEs but was blinded to their identities for the duration of the research. Our team’s prior involvement with the NPE program created both insight and risk of bias. To mitigate this, we used analytic memoing and regular reflexive discussion to challenge assumptions.

### 2.8 Ethical approval

Ethical approval for this study was granted by the Swansea University Medical School Research Ethics Sub-Committee (Approval Number: 3 2024 10355 10163).

## 3 Results

### 3.1 General information

A total of 132 NPEs were eligible to submit reflective essays across the two academic years. Eighty-two essays (2022/23 *n* = 39, 2023/24 *n* = 42) were submitted (response rate 62%). Six submissions were blank or non-serious and were excluded, resulting in 76 reflective essays included in the final analysis (inclusion rate 93% of submitted essays; 58% of eligible NPEs). A total of 23,587 words were analyzed of which 6,564 formed the basis of 182 coded entries that comprised two overarching themes. Table 2 illustrates the formation of themes from their respective codes and exemplar quotes.

**Table 2:** Overview of the emergent themes.

Theme and definition	Code	Description of code	Illustrative quotation
Cognitive Toolkit This theme encompasses the deliberate application of evidence-based strategies grounded in cognitive science. It refers to the specific, practical tools and techniques NPEs use to structure information, manage cognitive load, and enhance knowledge retention and understanding for their learners.	Retrieval Practice	The process of actively recalling information from memory, which strengthens neural pathways and improves long-term retention. This represents a shift from passive review to active, effortful learning.	*“I encouraged my students to incorporate spaced repetition into their revision methods, and tried to purposely use it in my workshops by including a quiz.” (2022/23 P21)*
	Dual Coding	The practice of presenting information in both verbal and visual formats simultaneously. This leverages separate cognitive channels to reduce extraneous load and create richer, more interconnected mental models of complex concepts.	*“I used the anatomy models and directly demonstrated the movements that the muscle performs. I tried to add onto this by providing a verbal commentary and drawing tables on the whiteboard to summarize information.” (2023/24 P31)*
	Concrete Examples	The strategy of reducing abstraction by linking theoretical or complex concepts to tangible, real-world, and often clinical scenarios. This makes knowledge more meaningful, memorable, and easier for learners to apply.	*“I opted to explain vascular territories of the brain and how their occlusion precipitates a stroke using a parallel example of motorways into a city being closed off, preventing food deliveries.” (2022/23 P9)*
	Instructional Scaffolding	The technique of providing temporary, structured support for a complex task and gradually withdrawing it as the learner’s competence grows. This keeps the learner in their zone of proximal development, ensuring the task is challenging but achievable.	*“I would build up on the concepts – start simple and build up slowly- and ask challenging questions which allowed students to make the leap to the next core concept.” (2023/24 P27)*
Humanistic Framework This theme encapsulates the pedagogical practices focused on the learner as an individual and the cultivation of a supportive, inclusive, and engaging learning environment. It refers to the relational and affective dimensions of teaching, prioritizing the learner’s experience and psychological wellbeing as a prerequisite for effective learning.	Universal Design of Learning Resources	The proactive planning and creation of teaching materials and activities that are accessible and usable by the widest possible range of learners from the outset, thereby respecting diversity in learning preferences and abilities.	*“I accessed a great scope of resources for sessions to cater to all individuals’ teaching needs, such as creating my own PowerPoints, requesting specific models, using a whiteboard for drawings and cadaveric materials. I try to use the principles of universal design when organizing these.” (2022/23 P14)*
	Adaptive Teaching	The dynamic, real-time adjustment of one’s teaching approach, content, or pace based on the learners’ engagement, understanding, and emergent needs. This demonstrates a high degree of responsiveness and learner-centeredness.	*“We had to learn how to adapt our teaching style to the engagement of our students and the pre-existing level of knowledge they had.” (2023/24 P4)*
	Active Learning	The use of instructional methods that intentionally involve students in the learning process through activities and discussion, requiring them to engage in higher-order thinking rather than passively listening.	*“I got students to make their own mini spotters on the cadavers to test each other.” (2023/24 P23)*
	Establishing Psychological Safety	The intentional creation of a low-stakes, non-judgmental atmosphere where learners feel secure enough to be vulnerable, ask questions, admit confusion, and make mistakes without fear of negative consequences to their self-worth.	*“I remember feeling not confident to volunteer ideas when I did this module, so I made real effort to reaffirm this is a space for learning, and discussed my own challenges with anatomy studies.” (2022/23 P29)*
	Collaborative Learning	The structuring of learning tasks to leverage social interaction, where students work together to achieve a shared goal. This promotes peer-to-peer discussion, co-construction of meaning, and a sense of shared community.	*“I told them to work together to complete each question on their worksheets, then I would take them in smaller groups to the cadavers where they had to collectively ‘teach’ me the anatomy relevant to their worksheets.” (2023/24 P18)*
	Formative Assessment of Teaching	The practice of an educator actively seeking feedback on their own teaching performance from learners or peers, with the goal of reflectively adapting and improving their pedagogical approach for future sessions.	*“I found that by asking learners for feedback during and after sessions, I was able to tailor following teaching elements to better suit their preferences and needs.” (2023/24 P24)*
	Checking for Understanding	The ongoing process of monitoring learner comprehension during a teaching session through questioning and observation, allowing the educator to identify and address misconceptions in real-time.	*“I tried to implement chunk and checking into my teaching sessions.” (2022/23 P16)*
	Experiential Learning	A process of learning through direct, hands-on experience, often involving physical interaction with learning objects or environments, grounded in the belief that ‘learning by doing’ creates more memorable understanding.	*“I felt it important that students had sufficient time with cadavers and models to enhance their learning. I would make them physically trace out the courses of major nerves and explain their motor and sensory functions as they moved through a cadaver head to toe.” (2023/24 P18)*

Table 3 demonstrates the 12 evidenced-based considerations for teaching practice which were elucidated from the submitted reflections. Out of these came two central, interconnected themes that together constitute a distinct pedagogical craft: the deliberate use of a Cognitive Toolkit to structure learning, and the cultivation of a Humanistic Framework to support the learner and inform their teaching approach.

**Table 3:** Codes and associated frequencies comprising the two main themes.

Theme	Code	Code frequency (*n*)	Reflection frequency (*n*)
Cognitive Toolkit	Retrieval Practice	16.5% (30)	36% (27)
	Dual Coding	10.4% (19)	22% (17)
	Concrete Examples	5.5% (10)	11% (8)
	Instructional Scaffolding	4.4% (8)	9% (7)
Humanistic Framework	Universal Design of Learning Resources	13.2% (24)	30% (23)
	Adaptative Teaching	12.1% (22)	24% (18)
	Active Learning	11.5% (21)	25% (19)
	Establishing Psychological Safety	11% (20)	20% (15)
	Collaborative Learning	6% (11)	14% (11)
	Formative Assessment of Teaching	3.8% (7)	8% (6)
	Checking for Understanding	2.7% (5)	5% (4)
	Experiential Learning	2.7% (5)	7% (5)
	TOTAL	182	–

### 3.2 The cognitive toolkit: applying the science of learning

A predominant narrative within the reflections was the conscious and strategic application of principles from cognitive science. This was not a haphazard use of “teaching tips,” but a deliberate effort to manage the high intrinsic cognitive load of anatomy. The tools within this kit were often used in concert, forming a logical instructional sequence. The process frequently began with Retrieval Practice as a strategy to move learners from a passive to an active stance by prompting recall of prior knowledge. NPEs described engineering these moments to activate thinking which overcome the initial inertia of a learning session. This was often followed by the use of Dual Coding, where educators would intentionally present new information in multiple formats. They described a dynamic process of verbally explaining a concept while simultaneously demonstrating it on an anatomical model or sketching it on a whiteboard. This multi-modal approach directly tackles the complex, spatial nature of the subject matter. The learning of this new information was then supported by Instructional Scaffolding, a method of providing temporary support which is gradually withdrawn as learners gain competence, thereby keeping the task challenging but not overwhelming as the educator structures the learning task to keep it within the learner’s zone of proximal development. Finally, these concepts were solidified through the use of Concrete Examples, which served to anchor the abstract anatomical facts in memorable clinical contexts. This toolkit represents a coherent pedagogical strategy: activate prior knowledge, present new knowledge in a rich and multi-modal format, and then ground it in a meaningful, applied context.

### 3.3 The humanistic framework: cultivating the learning environment

Running parallel to this cognitive focus was an equally powerful narrative centered on the holistic experience of the learner. This humanistic framework was concerned not with the mechanics of memory, but with creating the socio-emotional conditions under which learning could flourish. The foundation of this framework was the deliberate effort to Establish Psychological Safety. This was described not as a passive state of being friendly, but as an active pedagogical strategy to create a low-stakes environment where learners felt secure enough to be vulnerable, ask questions, and expose their misunderstandings. This principle was then put into action through a profound commitment to Adaptive Teaching, wherein NPEs consistently reflected on the need to be responsive and flexible, adjusting their approach in real-time based on the learners’ engagement, understanding, and energy levels. This bespoke approach demonstrates a deep respect for the individual learner’s journey. These efforts were often supported by Universal Design, where educators would proactively prepare a range of resources and teaching modalities to cater to the anticipated diversity of their groups. Their experiences were structured around Active, Experiential, and Collaborative Learning methods, shifting the focus from the teacher to the learner as an agent in their own education. This relational approach was reinforced through consistent feedback loops, such as Checking for Understanding and seeking Formative Assessment of their own Teaching, acts which build trust and position learning as a partnership. This framework, in its entirety, reveals a pedagogical philosophy that is deeply relational, empathetic, and learner-centric, recognizing that a learner’s emotional state is inseparable from their cognitive capacity.

## 4 Discussion

This inquiry identifies the GEM near-peer educator as a reflective practitioner engaged in a sophisticated pedagogical craft. Their approach is not monolithic but is characterized by a dynamic application of a scientifically informed Cognitive Toolkit with a deeply relational, Humanistic Framework. This duality of the commitment to both the science of learning and the art of creating a supportive environment, provides a rich ground for discussion when situated within the broader landscape of medical education.

### 4.1 Cognitive toolkit

The deliberate use of techniques comprising the Cognitive Toolkit by NPEs evidences their journey beyond intuitive or “common sense” approaches to teaching toward a more professionalized, evidence-based practice (27–29). The use of retrieval practice directly confronts the high-volume memorization demands of anatomy, aligning perfectly with a large body of literature demonstrating its superiority for long-term retention (30–34). Similarly, the frequent use of dual coding and instructional scaffolding shows an implicit understanding of cognitive load theory (33, 35). These NPEs appear to be actively managing the cognitive demands on their learners by presenting information in optimized formats and by structuring complex tasks to prevent overload (36–38). This application of theory to practice is the very essence of what Shulman (39) termed “pedagogical content knowledge”; conferring a capacity to transform subject matter expertise into a form that is digestible and accessible for a novice.

The clear influence of Swansea’s Clinical Educators Programme affirms the value of including formal pedagogical training in NPT programs. The techniques belonging to the Cognitive Toolkit were largely explored in the asynchronous teacher training module completed at the start of the NPE post. The longevity of the toolkit persisting within their reflections further evidences that brief, targeted teacher-training courses can embed core principles from educational theory into the self-reported practice of early-career educators (40, 41). If adopted more widely, such approaches may partially address long-standing concerns in the literature regarding the otherwise ad-hoc and informal nature of NPE training stemming from the absence of a centralized curriculum (15, 16). Our study proposes that such training is not only necessary but is eagerly adopted and applied by this more mature cohort of early career clinical educators.

### 4.2 Compassionate educators

The most profound insight arises from the Humanistic Framework that the NPEs appeared to construct independently. Their significant emphasis on psychological safety and adaptive teaching is a powerful finding. This focus on the implication of effect on learning within NPT is recently reported in literature (42, 43). The framework extends the foundational NPT theories of congruence. While Lockspeiser et al. (1) framed social congruence as an inherent, structural property of the near-peer dyad, our data suggests it is also something that these GEM NPEs actively and intentionally *construct*. The effort to be “approachable” and create a “safe space” appears to be a deliberate pedagogical act, a form of relational work aimed at operationalizing the theoretical benefits of social congruence (44). This reconceptualizes the NPE role, moving the educator from a simple beneficiary of a structurally congruent relationship to its primary architect. They are not just similar to their learners; they are actively leveraging that similarity to build trust and foster an environment conducive to learning while embracing vulnerability (3).

The NPEs’ commitment to Universal Design reflects a proactive ethos of inclusivity, planning for diversity before the session even begins (45, 46). This is complemented by the reactive, in-the-moment skill of Adaptive Teaching, which allows NPEs to tailor their approach to the emergent needs of the group (47). Crucially, this supportive environment was not passive. The frequent reports of Active, Collaborative, and Experiential Learning demonstrate a commitment to a constructivist pedagogy where learners are empowered as agents in their own knowledge creation (48–51). This active engagement is the very mechanism through which the benefits of Psychological Safety are realized; a space is safe not just for listening, but for trying, failing, and questioning (52). This dynamic was sustained through continuous feedback loops. The act of Checking for Understanding could be viewed as a relational gesture of care, while the open display of seeking Formative Assessment of their own teaching endeavors present the NPE not as an unassailable expert but as a co-learner, modeling the willingness to embrace vulnerability they seek to foster (53–56).

This active cultivation of a safe and responsive learning environment can be understood as an early and powerful form of professional identity formation (57). The humanistic values these NPEs enact in the classroom, namely empathy, responsiveness, and inclusivity are the very same values that underpin relationship-centered care in clinical practice (58, 59). In essence, the NPT experience serves as a rehearsal space where educators are not only refining how to think, act, and *feel* like a physician, but also expand on how to become a compassionate educator. For these NPEs, the reality of a disengaged or struggling student is a problem to be solved with responsive, humanistic engagement. Their use of adaptive teaching embraced a willingness to place learners at the heart of NPE approaches (47). Watling et al. (60) argue the requirement to interpret ambiguous cues and make credibility judgments inherent in adaptive approaches to teaching may also benefit educators in navigating the “messiness” of clinical reasoning in complex environments.

This relational work, however, also constitutes a form of unmeasured and often unacknowledged emotional labor (61). The significant effort invested in managing the learning environment and supporting the emotional needs of students is a critical component of NPEs success, yet it is rarely accounted for in institutional evaluations (62). If emotional labor predicts exhaustion and disengagement, as recently suggested by Zhai et al. (63) then NPE training programs ignoring this dimension risk promoting burnout and attrition. Indeed, while NPEs implemented evidence-based strategies, as some expressed uncertainty about their effectiveness or struggled with consistency. Thus, reinforcing their ongoing professional development opportunities will help them refine and sustain these practices.

### 4.3 Limitations

While this inquiry offers rich insights into the pedagogical philosophies of anatomical GEM NPEs, its findings must be interpreted in light of several important limitations. The primary limitation lies in the study’s reliance on self-reported data derived from reflective essays. This methodology is susceptible to a number of biases that temper the certainty of our conclusions. A significant gap can exist between a practitioner’s “espoused theory” and their “theory-in-use,” that is, what they say they do versus what they actually do under the dynamic pressures of a teaching environment may be different (64). Reflections are reconstructions of events, subject to recall bias, and may be influenced by a social desirability bias, where participants describe their actions in terms that align with perceived best practices. Furthermore, an educator’s claim to have established ‘psychological safety’ is an interpretation of their own actions, which may or may not align with an objective observer’s assessment or, more importantly, the learners’ subjective feelings of safety (65).

The study’s methodological design has implications for the validity and generalizability of its findings. The absence of data triangulation means data lacks the corroborating perspectives that could be yielded from inclusion of direct observation or learner feedback (66). Observation would have provided objective data on the fidelity and frequency of the reported techniques, while learner interviews would have offered crucial insight into the interpreted impact of these pedagogical choices. Furthermore, the findings are drawn from a single institution and a highly specific cohort: graduate-entry medical students who voluntarily engaged in a reflective exercise as part of a voluntary program. This cohort’s maturity and prior academic experience may foster a pedagogical sophistication not representative of all (particularly undergraduate) NPE populations. Due to the voluntary nature of NPE applications, it is plausible that this study captures the practices of the most engaged and reflective individuals, presenting a ‘best-case’ rather than a typical scenario of NPE practice.

### 4.4 Recommendations for near-peer educator training

#### 4.4.1 Promote a community of practice

Training must evolve from teaching a set of techniques to developing a way of thinking. Future programs should be designed as communities of practice, using case-based discussions and problem-based learning to explore complex pedagogical challenges. The goal should be to help NPEs articulate, question, and refine the sophisticated philosophies they are already developing, providing them with the language and theoretical frameworks to understand their own intuitive practices.

#### 4.4.2 Developing advanced relational and facilitative skills

The humanistic framework identified requires a high level of interpersonal skill. Educator training should incorporate practical, simulation-based workshops focused on advanced facilitation, active listening, providing constructive feedback, and managing challenging group dynamics. This would build the capacity to skilfully manage the affective and social dimensions of learning.

#### 4.4.3 Establishing a culture of longitudinal peer coaching

Professional growth is a continuous process. Training programs should be longitudinal, not “one-and-done.” After an initial foundation, the training focus should shift to a model of structured peer coaching, where NPEs observe one another teach and engage in reflective, non-judgmental debriefs. This institutionalizes reflective practice, fosters a collaborative culture, and provides the ongoing support needed to translate good intentions into consistent, high-quality teaching.

## 5 Conclusion

The graduate-entry NPE is not a novice simply repeating memorized facts. They are practitioners of a developing craft, striving to blend the rigor of learning science with a deeply humanistic commitment to their students. They are readily capable of applying taught cognitive tools while simultaneously and independently building a relational framework for learning. This recognition requires that we, as a medical education community, meet their sophistication with a commensurate level of support. By evolving our training models from simple instruction to the sustained cultivation of reflective practice within a community of peers, we can better support the development of these future clinical educators. In doing so, we not only enhance the quality of our NPT programs but also enrich the very culture of teaching and learning within the wider healthcare professions faculty.

## Data Availability

The raw data supporting the conclusions of this article will be made available by the authors, without undue reservation.
