# Supplementary material for: Pedagogy in practice: a qualitative analysis of evidence-based teaching methods used by graduate-entry near-peer medical educators
Source: Front Med (Lausanne). 2026 Feb 27;13:1757648. doi: 10.3389/fmed.2026.1757648 (PMC12982050; doi:10.3389/fmed.2026.1757648)
Supplement: Supplementary file 1 [file Data_Sheet_1.PDF]

frontiers

Supplementary Material

1 Clinical Educators Programme - Swansea University

The Clinical Educators Programme (CEP) is a non-credit baring asynchronous online module delivered via the virtual learning environment, Canvas. The programme is intended to be completed by near-peer clinical demonstrators employed at Swansea University as Senior Teaching Assistants. It is aligned with UKPSF framework to facilitate an application for Associate Fellow of the Higher Education Academy. There are four main learning outcomes of this module:

1. Identify some of the key evidence-based principles of 'how people learn'
2. Identify the essential actions required for an effective anatomy demonstration
3. Conduct a peer observation and review of a teaching session
4. Demonstrate an ability to evaluate and reflect on teaching to identify aspects for improvement

A breakdown of the mapping of these learning outcomes is contained in *Supplementary Table 1*.

Supplementary Table 1: Overview of structure of CEP

Phase	Focus	LO's	Core Topics	Delivery Format	Approx. Time (h)
1	Foundations of Practice	1, 2	Cognitive load theory, retrieval practice, dual coding, concrete examples	Videos, Papers, Quizzes	3.5
2	Peer Observation	3	Observation principles, feedback	Video, Reading, Structured form	2
3	Reflective Practice	4	Models of reflection, educator identity	Reading, Papers, Podcast, Prompts	3

Exemplar screenshots of the various landing pages for the CEP modules on Canvas are displayed in *Supplementary Figures 1-4* below.

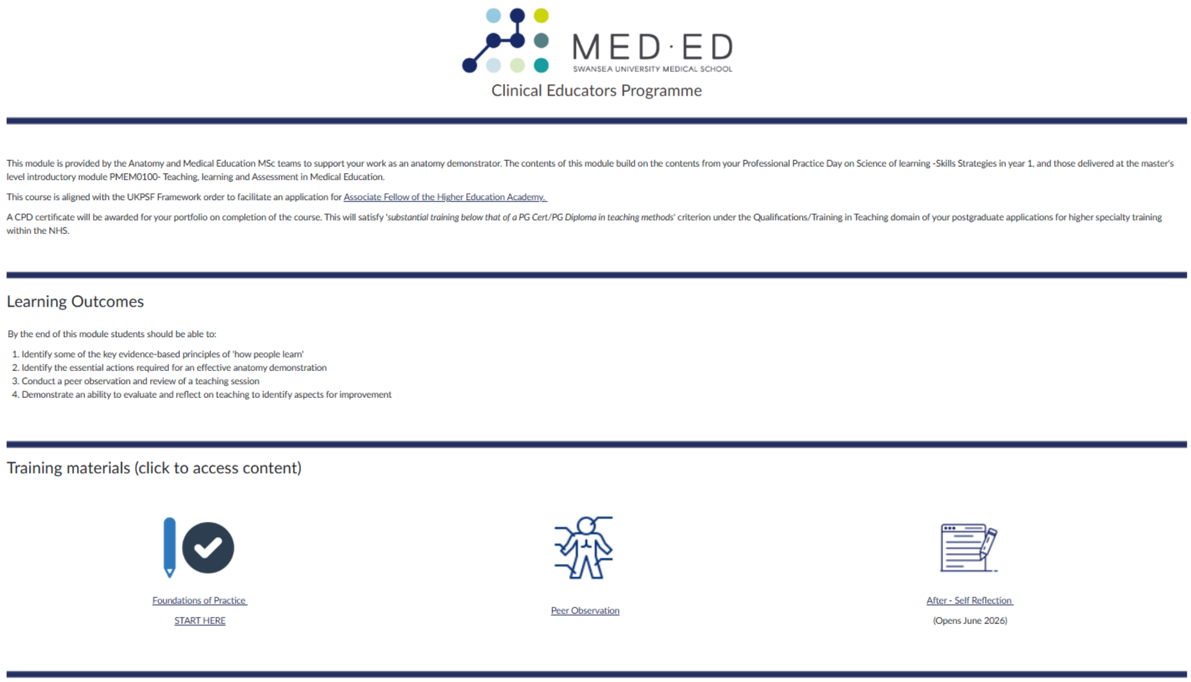

Supplementary Figure 1. *Screenshot of CEP landing page on Canvas, outlining learning objectives and programme structure. Image produced 1/2/2026.*

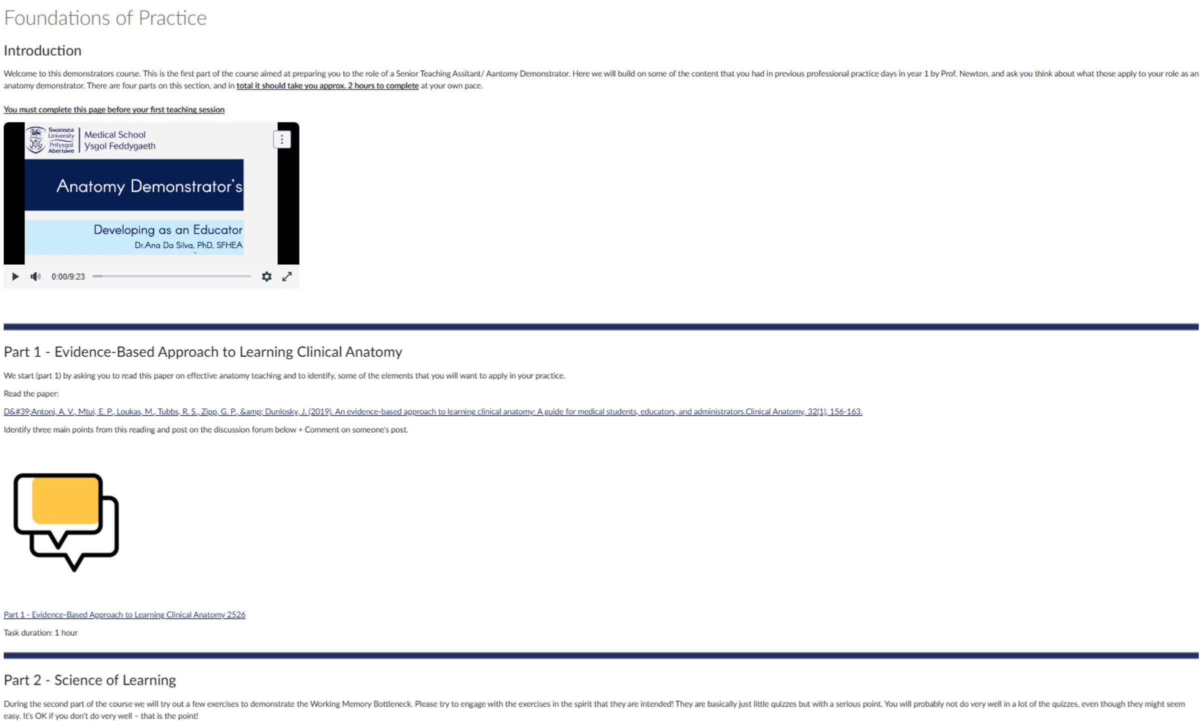

Supplementary Figure 2. *Screenshot of first part of CEP Phase 1, Foundations of Practice with introductory video, seminal paper review and learning theory talks. Image produced 1/2/2026.*

During - Peer Observation

Introduction

Welcome to the second section of the anatomy demonstrators course. This section is aimed at supporting your peer observation of teaching.

Observation is a critical skill in medicine and in education alike!

Peer observation of teaching is a valuable practice in the field of education that involves teachers observing and providing feedback to their colleagues. This process offers several benefits that contribute to professional growth, improved teaching practices, and enhanced student learning outcomes.

Firstly, peer observation fosters a culture of collaboration and professional development among teachers. It provides opportunities for teachers to learn from one another, share innovative ideas, and reflect on their own teaching methods. This collaborative approach helps create a supportive and constructive environment that promotes continuous improvement.

Secondly, peer observation encourages self-reflection and self-evaluation. By observing their peers, teachers can gain insights into their own teaching practices, strengths, and areas for improvement. Engaging in reflective discussions with colleagues allows them to critically analyze their instructional strategies, leading to enhanced teaching effectiveness.

Furthermore, peer observation promotes the sharing of best practices. Teachers can observe and learn from effective techniques employed by their peers, which can be adapted and implemented in their own classrooms. This cross-pollination of ideas and strategies contributes to professional growth and leads to a more dynamic and enriched teaching environment.

Moreover, peer observation provides valuable feedback and support. Teachers can receive constructive criticism and suggestions for improvement from their colleagues, helping them refine their teaching methods and address any challenges they may be facing. This feedback is often more comfortable and less intimidating compared to evaluations conducted by administrators, fostering a positive and growth-oriented atmosphere.

Finally, peer observation of teaching has been found to positively impact student learning outcomes. Research shows that when teachers engage in collaborative reflection and receive feedback from their peers, it results in improved instructional practices and student achievement.

In conclusion, peer observation of teaching offers numerous benefits including collaboration, self-reflection, sharing of best practices, feedback, and improved student learning outcomes. By implementing this practice, educators can create a supportive and effective learning community that facilitates professional growth and development.

References:

1. Bell, M. A., & Mladenovic, R. (2018). Peer observation: Learning from each other. Teaching and Learning in Nursing, 13(3), 198-201.
2. Campbell, M., Cavanagh, M., & Griffin, P. (2016). Investigating the impact of peer observation of teaching in nursing and midwifery education: A systematic review. Nurse Education Today, 41, 162-171.
3. Gosselin, K. P., & Wilson, E. (2018). Peer observation as a collaborative, learning experience: An examination of the impact on faculty teaching. The Journal of Continuing Education in Nursing, 49(6), 287-293.
4. Sullivan, M., & McCarthy, M. (2018). The impact of peer observation on teaching: A systematic review. Nursing Education Perspectives, 39(6), 334-339.

Part 1 - How to observe others

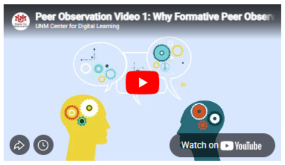

Useful reading:

Zarrin Seema Siddiqui, Diana Jonas-Dwyer & Sandra E. Carr (2007) Twelve tips for peer observation of teaching, Medical Teacher, 29:4, 297-300, DOI: 10.1080/01421590701291451

Part 2- Do it!

Supplementary Figure 3. *Screenshot of Screenshot of first part of CEP Phase 2, Peer Observation, with background reading instructions, video content and submission link (cropped). Image produced 1/2/2026.*

After - Developing Reflective Practitioners

Reflective Practitioners can be thought of as those who have mastered the skill of metacognition; thinking about thinking. They can recognize how and why they learn and identify gaps in their learning. They are able to use triggers and cues from their experience to prompt learning and build them into their metacognition. The evidence regarding the value of reflective practice is mixed, and in some ways it is difficult to even test. Yet reflective practice is a required part of most health professions, from students through to senior leaders, and thus we are required to develop reflective practitioners as part of our curricula.

This section is designed to give you the opportunity to reflect on your demonstrator experience and on what you learned from your peer-observation of other demonstrators.

Part 1 - Listen to our podcast Developing Reflective Practitioners with Prof Andy Grant on reflection in Medical Education

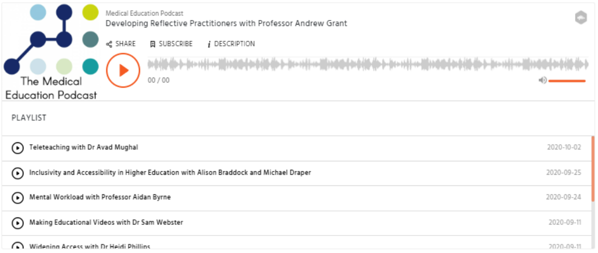

Part 2 - Reflect!

Please share with us a reflection on your teaching experiences as an anatomy demonstrator and on the observation you did of a peer demonstrator.

This is a 100% personal reflection, it will not be marked but will be used by us as evidence of course completion to issue your certificate.

Please submit a short reflection (between 250 words to 500 words) using the link below including, but not limited to, the following points:

1. Something that was difficult or/and unexpected and how you dealt with it
2. Something you have learned from being an anatomy demonstrator
3. Something that you learned from your peer observation of other demonstrators
4. Something you **do differently** in your future as a result of this experience (could be in medicine or in studying or even in your personal life)

Supplementary Figure 4. *Screenshot of first part of CEP Phase 3, Developing Reflective Practitioners, with podcast audio content, writing prompts and submission link (cropped). Image produced 1/2/2026.*
